# Effects of Hemin and Nitrite on Intestinal Tumorigenesis in the A/J Min/+ Mouse Model

**DOI:** 10.1371/journal.pone.0122880

**Published:** 2015-04-02

**Authors:** Marianne Sødring, Marije Oostindjer, Bjørg Egelandsdal, Jan Erik Paulsen

**Affiliations:** 1 Norwegian University of Life Sciences, Department of Food Safety and Infection Biology, PO Box 8146 Dep., 0033, Oslo, Norway; 2 Norwegian University of Life Sciences, Department of Chemistry, Biotechnology and Food Science, PO Box 5003, 1432, Ås, Norway; National Institute of Agronomic Research, FRANCE

## Abstract

Red and processed meats are considered risk factors for colorectal cancer (CRC); however, the underlying mechanisms are still unclear. One cause for the potential link between CRC and meat is the heme iron in red meat. Two pathways by which heme and CRC promotion may be linked have been suggested: fat peroxidation and N-nitrosation. In the present work we have used the novel A/J Min/+ mouse model to test the effects of dietary hemin (a model of red meat), and hemin in combination with nitrite (a model of processed meat) on intestinal tumorigenesis. Mice were fed a low Ca^2+^ and vitamin D semi-synthetic diet with added hemin and/or nitrite for 8 weeks post weaning, before termination followed by excision and examination of the intestinal tract. Our results indicate that dietary hemin decreased the number of colonic lesions in the A/J Min/+ mouse. However, our results also showed that the opposite occurred in the small intestine, where dietary hemin appeared to stimulate tumor growth. Furthermore, we find that nitrite, which did not have an effect in the colon, appeared to have a suppressive effect on tumor growth in the small intestine.

## Introduction

Colorectal cancer (CRC) is the third most common cancer worldwide, and accounts for a large number of deaths each year [1]. Although the cause of sporadic CRC is not clear, it appears that food and nutrition is closely related with both causation and prevention of this type of cancer [2]. Intake of red and processed meat has been linked to an increased risk of colorectal cancer; however, it is still unclear exactly how these are connected. The relationship between red meat and CRC is complex, and may depend on other variables besides the meat itself; heme iron or nitrite in meat, harmful intestinal microbiota, abnormally functioning digestive system, or an unbalanced diet composition [3,4]. Research suggests that heme iron in the meat may be the culprit, which may explain why the link between CRC and red meat is stronger than the link between CRC and intake of white meat, which is low in heme iron [5,6]. Two pathways by which heme and CRC promotion may be linked have been suggested: fat peroxidation which produces aldehydes capable of forming mutagenic adducts with DNA, and N-nitrosation which may result in the formation of N-nitroso-specific DNA adducts. The former pathway may explain CRC promotion by fresh, red meat, while the latter may clarify CRC promotion by nitrite-cured (processed) meat [7]. Heme iron is believed to catalyze the endogenous formation of N-nitroso compounds (NOCs) in the colon, many of which are known carcinogens [6,8]. In processed meat, added nitrate and nitrite may also contribute to exogenous formation of NOCs within the meat. Furthermore, nitrite cured meat combines heme iron and nitrite in the digestive tract producing more endogenous NOCs than fresh meat, which may account for the hypothesis that processed meat is more closely related to CRC promotion than fresh meat [2,9].

Development of colorectal cancer is a multistep process involving tumor initiation, promotion, and progression. In humans, most colorectal cancers progress very slowly; it may take 5–20 years from early colonic lesions to develop into benign adenomatous polyps, and another 5–15 years for those adenomas to develop into a malignant carcinoma [10,11]. Most cases of colorectal cancer appear to be caused by somatic mutations, but a small number of cases are the result of germ-line mutations in the tumor-suppressor gene adenomatous polyposis coli (APC) which causes an inherited condition called familial adenomatous polyposis (FAP) [12,13]. FAP is characterized by the development of multiple adenomas in the colon, and occurs when an individual that has inherited one mutated *APC* allele experiences loss of heterozygosity. Inactivation of the second *APC* allele causes decreased degradation of β-catenin and activation of the canonical Wnt signaling pathway, which in turn leads to dysplasia [14–16]. In individuals who do not have FAP, approximately 80% of sporadic colon cancer cases still appear to be related to mutations in the *APC* gene where one allele mutates first, followed by mutation in, or loss of, the second allele [17].

The APC multiple intestinal neoplasia (Min/+) mouse (*Mus musculus*) is one of the most widely used murine models for human FAP. The Min mouse has a heterozygous truncation mutation at codon 850 of the tumor suppressor gene *APC*. This mutation is analogous to the mutation seen in the human *APC* gene, and results in the spontaneous formation of several neoplastic lesions in the mouse intestines [16,18–20]. FAP patients usually develop hundreds to thousands of adenomas in the colon and rectum, while the conventional Min/+ mouse, bred on a C57BL/6J genetic background, mainly develops adenomas in the small intestine and only a few in the colon [13,21]. A new Min/+ mouse strain, established at the Norwegian Institute of Public Health, develops a much higher number colonic lesions as compared to the C57BL/6J Min/+ mouse, and may therefore be more suited as a model for human CRC [22]. These early colonic lesions are known as flat aberrant crypt foci (flat ACF), and are visible as enlarged crypts with compressed pit patterns, which are not elevated from the mucosa, and are only visible with methylene-blue staining and transillumination. The flat ACF exhibits a continuous development from the monocryptal stage to adenoma. The adenoma, a benign lesion that may develop into a malignant adenocarcinoma, resembles the flat ACF, but contains a larger number of aberrant crypts, and is usually elevated from the mucosa [23].

The objective of the present study was to investigate the potential involvement of dietary heme and dietary nitrite on the development of colorectal cancer in the A/J Min/+ mouse model.

## Materials and Methods

### Ethics Statement

Experiments were conducted in accordance with The Norwegian Regulation of Animal Experimentation, and approved by the Norwegian Animal Research Authority (application ID: 5556). All animals were sacrificed by cervical dislocation.

### Animals

The A/J Min/+ mouse was produced at the Norwegian Institute of Public Health after backcrossing the Min/+ trait onto an A/J genetic background for more than 12 generations to secure their status as inbred [22]. This mouse was transferred to the Norwegian University of Life Sciences, Campus Adamstuen, where it has been maintained for several generations. The mice are maintained as an inbred colony, with brother-sister breeding as the chosen breeding-pair configuration when possible. New A/J blood is regularly added to the colony by backcrossing A/J +/+ females purchased from Jackson Laboratory (The Jackson Laboratory, Bar Harbor, ME) with resident A/J Min/+ mice in order to uphold the A/J Min/+ mouse line. All animals involved in the present study were bred at the experimental animal facilities at the Norwegian University of Life Sciences, Campus Adamstuen.

A/J Min/+ males were mated with A/J +/+ females, and the resulting Min/+ pups from each litter were used for the experiment. In total, 80 Min/+ offspring were used (1:1 ratio of males to females). All animals, both parents and offspring, were housed in Makrolon Type III open top plastic cages in a room with a 12-hour light/dark cycle, 55–65% humidity and 20–22°C. Water and feed were given *ad libitum*. Once born, the litter remained in the parental cage until weaned at 19–21 days, after which the pups were separated from the parental cage. Only mice with the APC mutation were used for the experiments, and to correctly determine the genotype of each mouse, ear tissue was collected at time of weaning. The ear tissue was subsequently processed to extract DNA for polymerase chain reaction. Allele-specific PCR was used to genotype the mice using three primers; MAPC MT (5’-TGAGAAAGACAGAAGTA-3’), MAPC 15 (5’-TTCCACTTTGGCATAAGGC-3’), and MAPC 9 (5’-GCCATCCCTTCACGTTAG-3’). The PCR product from a Min allele is 327bp long, while the PCR product from a wild-type allele (+) is 618bp long [24].

### Diets and experimental study design

Four experimental diets were designed: Hemin (model of red meat), Hemin+Nitrite (model of processed meat), Nitrite, and Control. The amount of hemin added to the diet was chosen based on demonstrated effectiveness in previous studies [25–28]. The Hemin+Nitrite and the Nitrite diet contained 2.8μmol/g of sodium nitrite (NaNO_2_). To balance the iron and sodium contents of the diets, each diet received the appropriate amount of iron (FeCl_3_) and/or sodium (NaCl): 0.5μmol/g and 2.8μmol/g respectively. Each experimental diet was on a semi-synthetic AIN-93M basis, with reduced calcium (15μmol/g) and no added vitamin D (0IU/g) (SDS special diet services, Witham, UK). To achieve low vitamin D, the milk protein casein, usually present in the AIN-93M diet, was exchanged for Hamlet soy protein for all four diets. The semi-synthetic AIN-93M diet base contained 4% fat and 73% carbohydrates. Both the Hemin and the Hemin+Nitrite diets contained 0.5μmol/g hemin, a ferric form of heme iron with a chloride ligand. Once genotyped, the A/J min/+ mice were randomly assigned to four experimental groups to test effects of the four diets on intestinal tumorigenesis: Hemin (n = 21, 11 females and 10 males), Hemin+Nitrite (n = 20, 10 females and 10 males), Nitrite (n = 20, 10 females and 10 males), and Control (n = 19, 10 females and 9 males). The animals were fed the experimental diets for eight weeks, from weaning at 3 weeks until 11 weeks, when the experiment was terminated.

### AIN-93M versus RM1

To demonstrate the dynamics of the novel A/J Min/+ mouse model, a parallel group of 15 A/J Min/+ mice fed the standard rodent diet, RM1, was included in the study. RM1 is a natural ingredient diet containing wheat, wheatfeed, barley, whey powder, soya oil, soya protein concentrate, and de-hulled extracted toasted soya. In contrast, the semi-synthetic AIN-93M diet used in this study contained Hamlet soy protein, corn starch, cellulose, sucrose, and soya oil. Both diets are supplemented with vitamins and minerals. Proximal analyses of both diets, based on values provided by the manufacturer (SDS special diet services, Witham, UK), are presented in S1 Table. Lesion development in 11 week (n = 9) and 25 week (n = 6) old RM1-fed A/J Min/+ mice was then compared to 11 week old mice fed the AIN-93M Control diet (n = 19).

### Scoring of small intestinal and colonic lesions

Mice were terminated by cervical dislocation at 11 weeks before the small and large intestines were excised from anus to stomach, rinsed in, and flushed with, ice-cold phosphate buffered saline solution (PBS), before being slit open longitudinally. The small intestines were divided into three parts; proximal, middle, and distal, while the colon remained intact. Both small and large intestines were then fixed flat between PBS-soaked filter papers. The flat fixed intestines were stored in 10% neutral buffered formalin for no less than 24 hours. Once fixated, the intestines were stained for 10–15 seconds with 0.2% methylene blue dissolved in the same formalin solution. The intestines were rinsed in fresh formalin to remove excess methylene blue stain, and stored in 10% neutral buffered formalin for another 24 hours, prior to examination by transillumination in an inverse light microscope. All examinations were done by an observer blind to the treatment of each sample. Surface microscopy was used to detect tumors as well as the early colonic lesions, flat ACF. The latter can be recognized by the difference in color as compared to normal epithelia; flat ACF stain a brighter blue/green while normal crypts a more subdued brownish-green. Furthermore, the flat ACF has enlarged crypts, lays flat against the surrounding epithelium, and has compressed luminal opening which give the lesion a gyrus-like appearance. The tumor resembles the flat ACF, but contains 30 or more aberrant crypts, and is usually elevated from the mucosa (S1 Fig). To describe the distribution of flat ACF and tumors, the recorded lesions were grouped into lesion size classes. The smallest colonic lesions observed ranged in size from 0.002 to 0.008 mm^2^, therefore this was chosen as the first size class. The remaining lesions were grouped into four additional size classes based on a suitable logarithmic scale with a base of 8: 0.009 to 0.064 mm^2^, 0.065 to 0.512 mm^2^, 0.513 to 4.096 mm^2^, and lesions greater than 4.097 mm^2^.

### Statistics

All results are expressed as mean values with 95% confidence intervals. All comparisons were performed two-tailed with a significance level of 5%. To test for differences between effects of the four diets, a two-way Analysis of Variance (ANOVA), with gender as a covariate, was used. Some gender differences were noticed, but no interactions between diet and gender were found, so the effects seen in the different diet groups were never dependent on gender. Thus, gender was pooled for further analysis. To test for differences between Hemin+ and Hemin-, and Nitrite+ and Nitrite-, a two-way ANOVA, was performed. If significant results were obtained in the two-way ANOVAs, the Holm-Sidak pairwise multiple comparisons post hoc test was used.

## Results

To determine the tumorigenic potential of the dietary interventions the following variables were scored: number of small intestinal and colonic tumors, number flat ACF in the colon, lesion size (mm^2^) and load (total area of the lesions).

### Body weight

Mice were fed specialized diets for 8 weeks, from weaning at week 3 till termination at week 11. After 8 weeks on the diets, there was a significant difference in body weight for animals in the four diet groups. Significant differences were also detected between males and females within each diet, however, no interaction between gender and diet was observed (Table 1). The Holms-Sidak post hoc test showed that animals in the Hemin+Nitrite group had a significantly lower body weight at termination than animals in both the Nitrite and the Control diet groups. However, there was no significant difference in the body weight of animals in the Hemin +Nitrite group when compared to those in the Hemin group. The animals fed Hemin were also significantly lighter than the control animals, but no such difference was observed when these animals were compared to animals fed nitrite alone. There was no significant difference in body weight detected in animals in the Nitrite diet group as compared to control animals.

**Table 1:** Average body weight of animals in a diet group, recorded after termination at 11 weeks.

Diet	Gender	N	Body weight
**Control** ^**a**^	Female	10	20.8 [20.1–21.5]
	Male	9	25.2 [23.8–26.6]
	Total	19	22.9 [21.6–24.2]
**Hemin** ^**bc**^	Female	11	19.6 [18.3–20.8]
	Male	10	22.0 [20.7–23.3]
	Total	21	20.7 [19.7–21.7]
**Hemin+Nitrite** ^**c**^	Female	10	18.2 [17.2–19.2]
	Male	10	21.4 [20.4–22.4]
	Total	20	19.8 [18.8–20.8]
**Nitrite** ^**ab**^	Female	10	20.3 [18.9–21.6]
	Male	10	23.4 [22.3–24.4]
	Total	20	21.8 [20.7–22.9]

All values are presented as mean [95% confidence interval]. Treatment means with a different superscript are significantly different at p<0.05. Two-way ANOVA p-values: Diet p<0.001; Gender p<0.001; Diet X Gender p = 0.410.

### Colon

**Fig 1:**
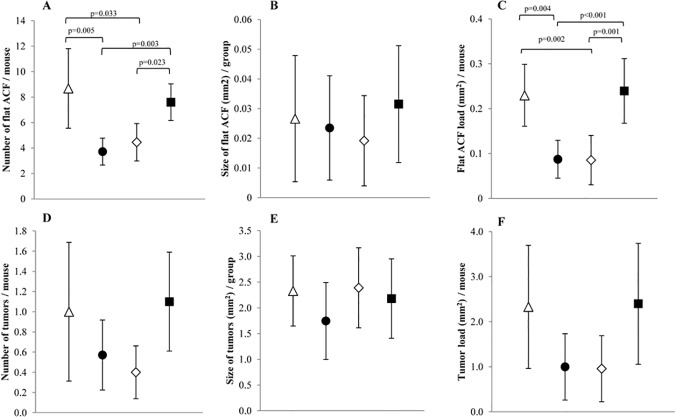
Colonic lesion development in A/J Min/+ mice fed four different diets; (△) Control; (●) Hemin; (◇) Hemin+Nitrite; (■) Nitrite. A-C shows data for flat ACF, while D-F presents data for colonic tumors. A and D) Number of lesions, B and E) size of lesions, C and F) flat ACF load and tumor load, respectively. Values represent the mean, error bars show the 95% confidence interval. Horizontal bars indicate significant difference between the groups.

In the colon, two types of lesions were observed; flat ACF and tumors (Fig 1 and S1 Fig). The number of flat ACF observed in the colon of mice fed diets Hemin or Hemin+Nitrite was significantly lower than in mice fed Nitrite or Control. The size of flat ACF did not differ between mice fed the four different diets. Nevertheless, a tendency for slightly smaller flat ACFs was seen in both Hemin and Hemin+Nitrite fed animals, although not significant. The total area of flat ACF per mouse (flat ACF load) was significantly lower in mice fed Hemin or Hemin+Nitrite than in mice fed Nitrite or Control. When compared with flat ACF, the mice had few colonic tumors. Interestingly, the patterns of tumor number (p = 0.090), tumor size and tumor load (p = 0.075) observed in animals fed the four experimental diets were similar to those observed for flat ACF. However, the differences in tumor scores did not achieve statistical significance.

**Fig 2:**
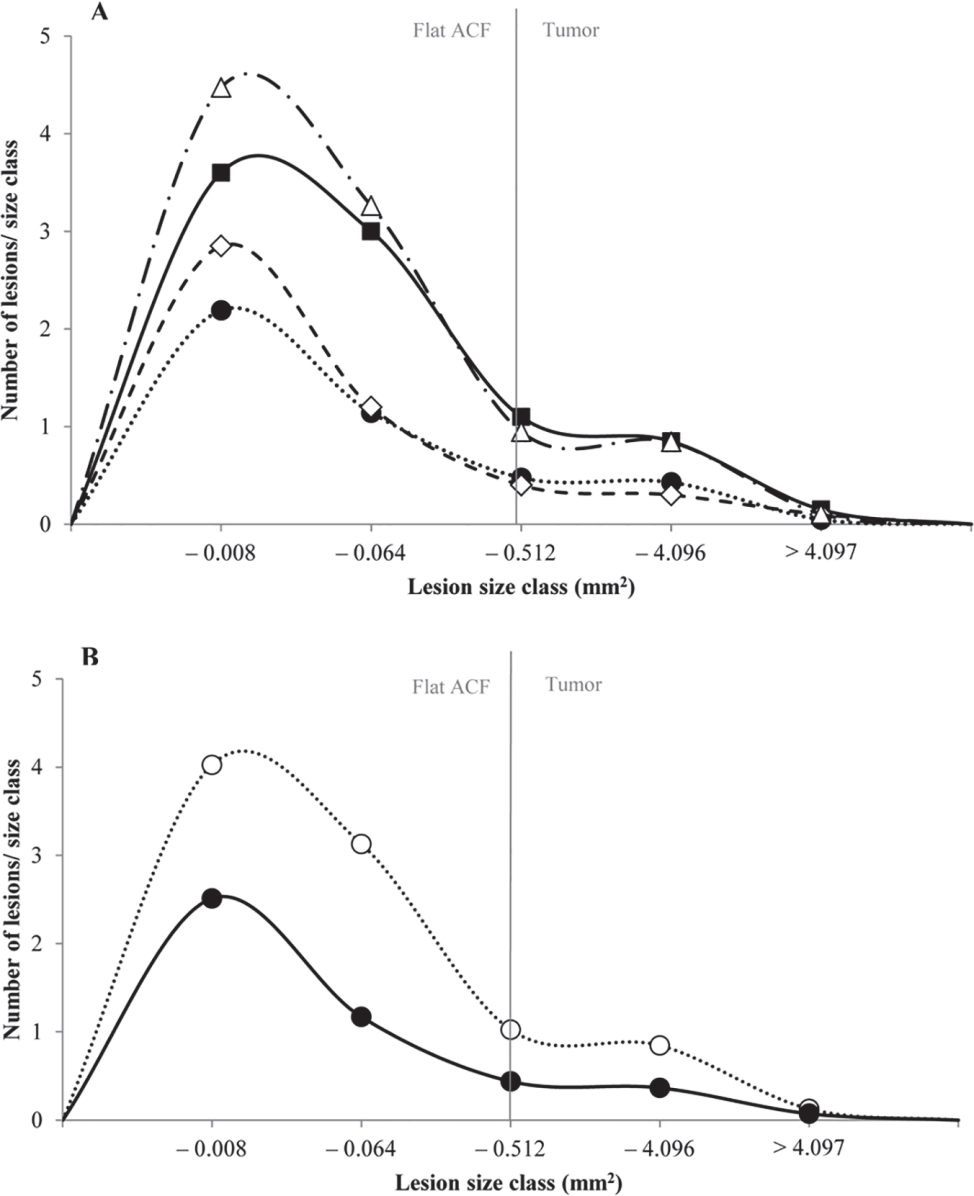
Size distribution of flat ACF and tumors in the colon of A/J Min/+ mice. A) mice fed four different diets, (△) Control; (**●**) Hemin; (**◇**) Hemin+Nitrite; (**■**) Nitrite, and B) mice fed diets with or without hemin, (**●**) Hemin^+^; (○) Hemin^-^. The smallest size class contained lesions with 1–4 crypts. One crypt had an area of 0.002mm^2^.In the colon, lesions are considered tumors if they contain more than 30 crypts/lesion (approximately 0.4mm^2^).

In previous studies, a continuous development from the monocryptal flat ACF to the stage of adenoma has been demonstrated; all lesions were characterized by severe dysplasia, altered control of β-catenin and rapid growth [29]. Hence, we present size distributions of pooled flat ACF and tumors to illustrate the effects of the dietary interventions (Fig 2). As expected, Hemin, or Hemin+Nitrite caused a shift towards smaller lesion sizes and fewer lesions compared with Nitrite or Control (Fig 2A). This suppressive effect was apparently due to the presence of hemin in the diet, as illustrated by the size distributions (Fig 2B) of these lesions in mice fed Hemin^+^ (pooled Hemin and Hemin+Nitrite) compared with mice fed Hemin^-^ (pooled Nitrite and Control). The Two-Way ANOVA (Table 2) showed that hemin in the diet (Hemin^+^) caused a statistically significant reduction in the formation of flat ACF and tumors as well as in the growth of flat ACF. Typically, the load of flat ACF and tumors was reduced by approximately 60% (p<0.001 and p = 0.019, respectively). When comparing mice fed Nitrite^+^ (pooled Nitrite and Hemin+Nitrite) to mice fed Nitrite^-^ (pooled Control and Hemin), no significant difference was observed (Table 2), indicating that nitrite in the diet did not have an effect in the colon of the A/J Min/+ mice.

**Table 2:** Number of lesions, lesions size, and lesion load in the colon and small intestine of A/J Min/+ mice fed a diet with or without hemin (*Hemin* ^*+*^: Hemin and Hemin+Nitrite; *Hemin* ^*-*^: Nitrite and Control), or a diet with or without nitrite (*Nitrite* ^*+*^: Nitrite and Hemin+Nitrite; *Nitrite* ^*-*^: Hemin and Control).

		Number of lesions/mouse		Lesion size (mm^2^/group)		Load (mm^2^/mouse)	
***Colon***		Flat ACF	Tumor	Flat ACF	Tumor	Flat ACF	Tumor
**Hemin**	Hemin ^+^	**4.07 [3.18–4.97]**	**0.49 [0.27–0.71]**	**0.02 [0.01–0.03]**	2.00 [1.47–2.54]	**0.09 [0.05–0.12]**	**0.98 [0.46–1.49]**
	Hemin ^-^	**8.13 [6.45–9.81]**	**1.05 [0.64–1.46]**	**0.03 [0.01–0.04]**	2.25 [1.74–2.76]	**0.24 [0.19–0.28]**	**2.36 [1.42–3.31]**
	P-value	**<0.001**	**= 0.020**	**= 0.023**	= 0.598	**<0.001**	**= 0.019**
**Nitrite**	Nitrite ^+^	6.03 [4.90–7.15]	0.75 [0.45–1.05]	0.03 [0.01–0.04]	2.24 [1.70–2.77]	0.16 [0.11–0.21]	1.68 [0.89–2.46]
	Nitrite ^-^	6.08 [4.33–7.82]	0.78 [0.40–1.15]	0.03 [0.01–0.04]	2.10 [1.60–2.60]	0.16 [0.11–0.20]	1.63 [0.86–2.40]
	P-value	= 0.418	= 0.881	= 0.831	= 0.559	= 0.930	= 0.942
**Hemin X Nitrite**	P-value	= 0.918	= 0.570	= 0.140	= 0.279	= 0.646	= 0.819
***Small intestine***							
**Hemin**	Hemin ^+^		22.05 [19.46–24.64]		**0.63 [0.45–0.80]**		13.80 [11.32–16.28]
	Hemin ^-^		20.43 [18.36–22.51]		**0.50 [0.38–0.63]**		10.31 [9.05–11.57]
	P-value		= 0.346		**<0.001**		ns (p = 0.084)
**Nitrite**	Nitrite ^+^		21.33 [18.98–23.67]		**0.54 [0.40–0.67]**		11.41 [9.58–13.24]
	Nitrite ^-^		21.20 [18.80–23.60]		**0.60 [0.43–0.77]**		12.79 [10.52–15.06]
	P-value		= 0.898		**= 0.024**		= 0.456
**Hemin X Nitrite**	P-value		= 0.208		= 0.055		= 0.999

Data is presented as mean [95% confidence interval]. Significant results are shown in bold text

### Small intestine

**Fig 3:**
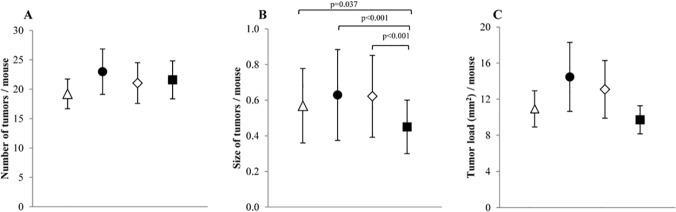
Small intestinal lesion development in A/J Min/+ mice fed four different diets; (△) Control; (●) Hemin; (◇) Hemin+Nitrite; (■) Nitrite. A) number of tumors, B) tumor size, and C) tumor load in the small intestine. Values represent the mean, error bars show the 95% confidence interval. Horizontal bars indicate significant difference between the groups.

**Fig 4:**
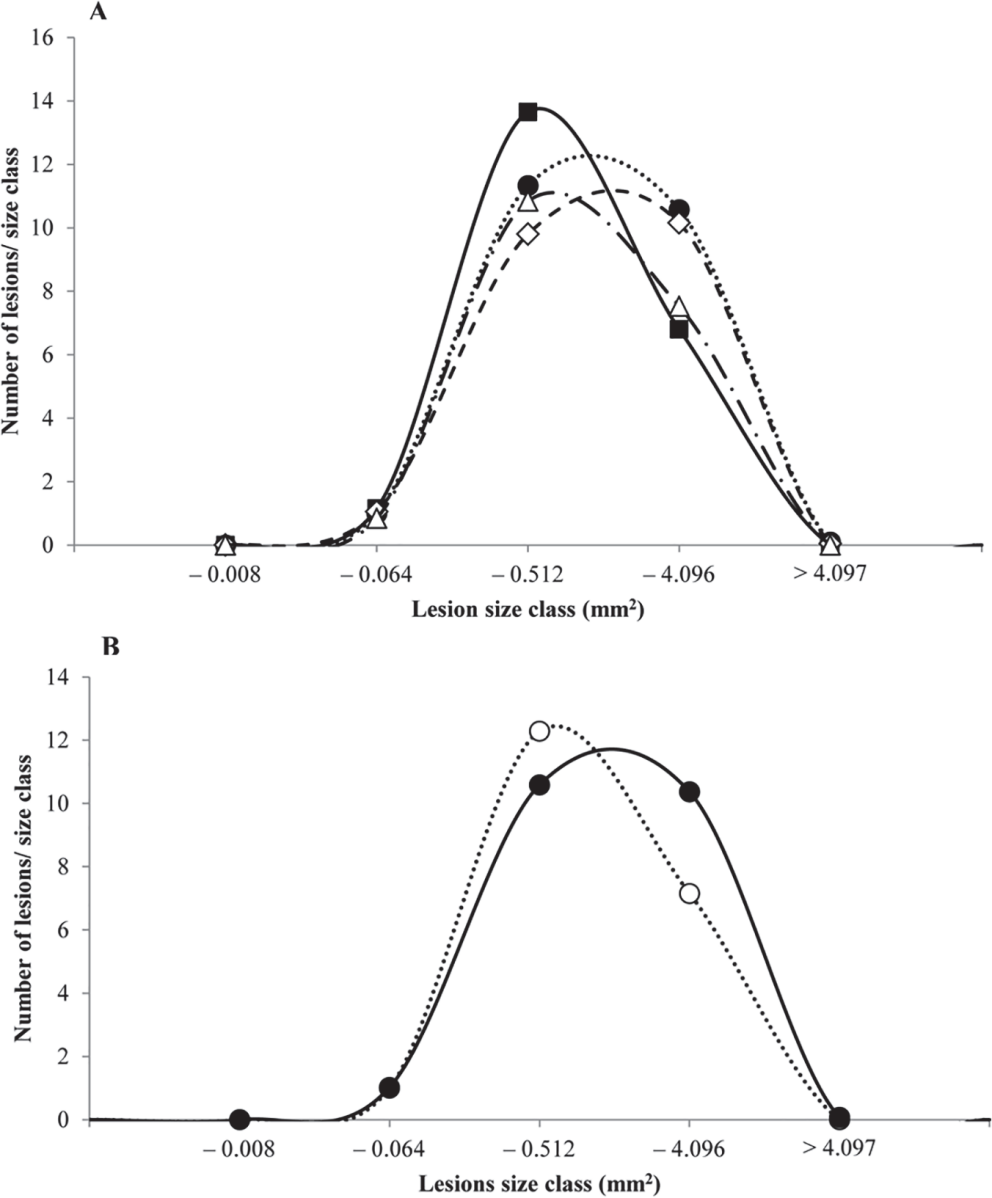
Size distribution of tumors in the small intestine of A/J Min/+ mice. A) mice fed four different diets, (△) Control; (●) Hemin; (◇) Hemin+Nitrite; (■) Nitrite, and B) mice fed diets with or without hemin, (●) Hemin^+^; (○) Hemin^-^. The smallest size class contained lesions with 1–4 crypts.

The size of small intestinal tumors was reduced in animals in the Nitrite group compared with animals in the Hemin, Hemin+Nitrite, or Control groups (Fig 3). However, the number of tumors, or tumor load did not differ significantly among animals fed the four diets. The mean sizes (Fig 3B), as well as the size distributions of the small intestinal tumors from animals (Fig 4A) fed these diets suggest that dietary hemin might stimulate tumor growth in the small intestines. This is further illustrated by the size distribution of small intestinal tumors from animals fed Hemin^+^ and Hemin^-^ (Fig 4B) and their differences in calculated tumor size (P<0.001; Table 2). Also a suggestive increase (P = 0.084) in tumor load (mm^2^/mouse) was observed in mice fed Hemin^+^ as compared with mice fed Hemin^-^ (Table 2). In contrast, the mean size of tumors in mice fed Nitrite^+^ significantly decreased when compared to mice fed Nitrite^-^, suggesting that dietary nitrite may cause a suppressive effect on tumor growth in the small intestine (Table 2).

### Comparison of control diet and basic maintenance diet

**Fig 5:**
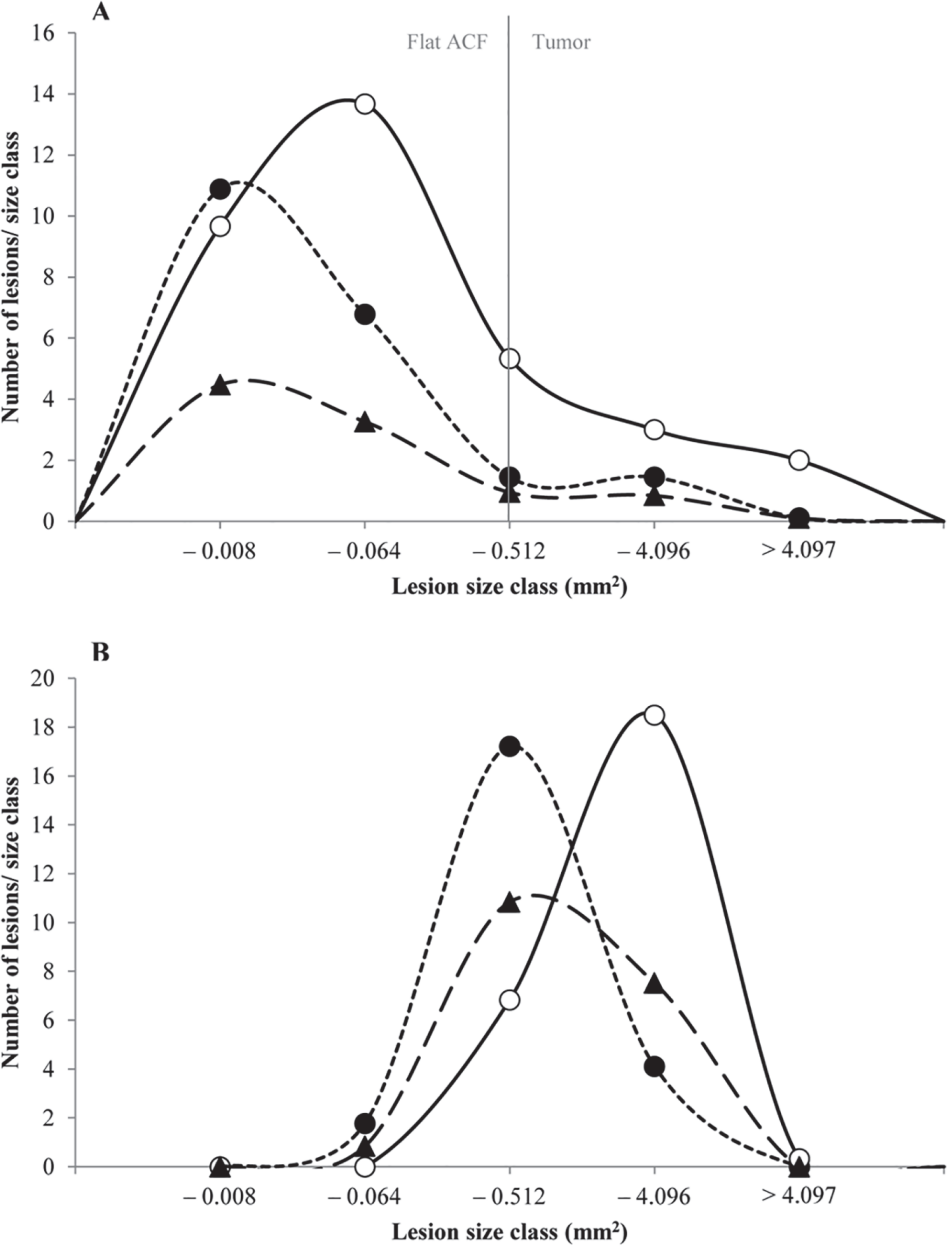
Size distribution of colonic and small intestinal lesions of A/J Min/+ mice fed two different rodent diets. A) flat ACF and tumors in the colon, and B) tumors in the small intestine of animals fed (▼) AIN-93M for 11 weeks, (●)RM1 for 11 weeks, or (○) RM1 for 25 weeks. The smallest size class contained lesions with 1–4 crypts. One crypt had an area of 0.002mm^2^. In the colon, lesions are considered tumors if they contain more than 30 crypts/lesion (approximately 0,4mm^2^).

The tumorigenesis induced by the Control (modified AIN-93) diet was compared with the tumorigenesis induced by the standard maintenance diet RM1 (Fig 5). In the colon, the RM1 diet yielded significantly more flat ACF (p<0.001) and more tumors than the Control diet, demonstrating that dietary components actually may stimulate the A/J Min/+ mouse model used (positive control). Furthermore, the size distribution data showed that flat ACF increased their size from week 11 to week 25, indicating a progression from early lesion to tumor. In the small intestine, animals fed RM1 for 11 weeks had a larger amount of lesions than 11 week old AIN-93M-fed animals. However, the AIN-93M lesions were significantly larger than those seen in RM1 animals of the same age (p<0.001). RM1-fed animals terminated at week 25 had a comparable number of lesions to those observed in the 11 week old mice, but the lesions in the 25 week old mice were significantly larger (p<0.001).

## Discussion

In the present experiment, the A/J Min/+ mouse was used to test the effect of dietary hemin (a model of red meat), and hemin in combination with nitrite (a model of processed meat) on intestinal tumorigenesis. Surprisingly, we found that mice fed hemin, either in combination with nitrite or not, experienced a significant decrease in both number and load of the early colonic lesions; flat ACF. Hemin thus appeared to suppress growth of flat ACF in the colon of A/J Min/+ mice. The iron(III) chloride added to the Control and Nitrite diets to balance for the heme iron in the Hemin and Hemin+Nitrite diets could potentially account for the differences observed in mice fed diets with or without hemin, however, from the literature it seems that the free iron concentration added to the non-hemin diets was too low to explain these tumors [30–34]. The decrease in number and load of flat ACF in A/J Min/+ mice fed hemin was mirrored for colonic tumors as well, albeit not significantly. We also saw a tendency for hemin-fed animals to have smaller colonic lesions than animals not fed hemin. Winter *et al*. [35] found that mice fed 0.2 μmol/g hemin showed an increase in proliferation when fed hemin for 3 weeks. Furthermore, they saw that apoptosis was inhibited in animals fed hemin for 6 months, and also that long term hemin-feeding increased DNA adducts in the colon. However, they did not see any increase in colonic neoplasms. This is similar to what we found in the present study.

Why hemin-fed A/J Min/+ mice in this study did not show an increase in colonic lesions is uncertain. It has previously been shown that anti-inflammatory drugs decrease the number of tumors in C57BL/6J Min/+ mice [36,37]. Thus, it could be speculated that the effects observed in the present study may, in some way, be connected to inflammation. Zhong *et al*. [38] found that, in a DSS–induced murine model for colitis, hemin decreased colonic inflammation, as well as reduced expression of the proinflammatory cytokine interleukin-17 (IL-17), which has been implicated in CRC [38–40]. The observed effect in the colon of hemin-fed A/J Min/+ mice in the present study may therefore be the result of a potential indirect anti-inflammatory effect of hemin.

In many animals studies investigating the effect of red and processed meat, fat is added to better resemble a westernized diet. One potential reason for the observed decrease in tumorigenic effect of dietary hemin in the colon of mice in this study is the low fat content in the diet. In the present study, no additional fat was added to the diet as our main objective was the potential effect of hemin; our special diets only contained the fat supplied in the base-diet AIN-93M which is 4% soy oil [41]. Intake of polyunsaturated fatty acids together with heme iron may lead to formation of peroxyl radical species in the intestinal tract, and consequently colorectal cancer [42]. Heme catalyzes the formation of reactive oxygen species (ROS), which leads to elevated levels of lipid peroxidation end-products such as malondialdehyde (MDA) and 4-hydroxynonenal (4-HNE), both of which are known risk factors for various diseases, including chronic inflammation and various cancers [7,43–45].

Ijssennagger *et al*. [25] found that heme has two separate effects on colonic epithelium; an acute reactive oxygen species stress and a delayed cytotoxicity stress. The delayed effects of heme caused cytotoxicity, hyperproliferation, and hyperplasia in the colon. Acute ROS stress induced the formation of lipid peroxidation products, and was evident shortly after heme intake, but no indication that ROS stress directly causes colonic hyperproliferation was found. However, intake of heme may lead to the formation of a cytotoxic heme factor (CHF), a compound thought to be formed when reactive lipid peroxides covalently bind to the protoporphyrin ring of the heme [46]. Cytotoxic heme factor formation is highly dependent on the presence of lipid peroxides, and, when enough accumulates in the colon, the cytotoxicity of the luminal contents increases, which in turn damages the colonic mucosa, and leads to hyperproliferation and hyperplasia [25]. In the present study, we observed a decrease in colonic lesions in animals fed hemin, and although this differs from other studies, it may possibly be because we did not add extra fat to the diet to bring about heme-induced ROS, and consequently formation and accumulation of cytotoxic heme factor.

Most studies investigating the effect of dietary heme add an additional amount of fat to the experimental diet, often 40% fat, to simulate the amount of a typical westernized diet, and these studies observe an increased risk of CRC [25–27,47–49]. However, there are a few studies in which less than 40% fat has been added to the experimental diet, and these studies show a somewhat similar tendency to what is seen in the present study. For example, neither Pence *et al*. [50] nor Lai *et al*. [51] observed an increase in colonic tumors in carcinogen-treated rats fed beef with a fat content of 5% and 6.35%, respectively. Pierre *et al*. [28] found that rats fed diets with 0.5 μmol/g hemin, in combination with 5% safflower oil, did not differ significantly from the control animals in the number of lesions recorded. The aforementioned, in combination with the results from the present study, may suggest that heme iron, which has been shown to be a CRC risk factor, may in fact require other dietary factors such as dietary fat, to be present, to cause cancer.

In contrast to results observed in the colon of the A/J Min/+ mice, lesions in the small intestine of animals fed hemin (Hemin^+^: Hemin and Hemin+Nitrite), were on average larger than lesions observed in animals not fed hemin (Hemin^-^: Nitrite and Control), which correlates with previous research with rodent models showing an increased risk of CRC with intake of dietary heme [26,48,52], however most of these studies are mainly conducted in the colon. In addition, we found that mice fed diets containing nitrite experienced a decrease in the size of small intestinal lesions, indicating a potential protective effect of nitrite on small intestinal tumor promotion. It is unclear why nitrite seemed to have a protective effect in the small intestine of A/J Min/+ mice, but it may be due to nitric oxide (NO) derived from dietary nitrite in the feed. NO is a signaling molecule that can regulate a variety of biological processes, and it has been suggested that dietary nitrite may have an anti-inflammatory effect [53,54]. NO may also possibly increase the amount of small intestinal mucus, which could likely explain the protective effect observed; the mucous layer in the intestine plays many important roles, including being the first line of defense against detrimental factors from the outside environment, such as those from foods [55,56].Van Hecke *et al*. [57] found that nitrite-cured pork meat inhibited both lipid and protein oxidation in *in vitro* digestion studies; however, at higher fat contents (20%), this inhibitory effect was less evident [57]. Also, Chenni *et al*. [9] found that nitrite given via drinking water reduced heme-induced lipid peroxidation in the colon of rats; however, this effect was not observed to be significant at lower doses.

During this study, a parallel group of A/J Min/+ mice were fed the standard rodent diet, RM1. These mice were included purely as a positive control to be compared to the control animals fed the AIN-93M special diet, and thus to demonstrate the effectiveness of the novel A/J Min/+ mouse model. The results indicate that animals fed RM1 had both larger and a higher number of colonic lesions than animals fed the AIN-93M control diet, which is consistent with previous results seen in C57BL/6J Min/+ mice, and likely attributed to compositional differences in these two diets, such as fiber, fat, and carbohydrate content [58].

After 8 weeks on the special diet, mice fed hemin or hemin in combination with nitrite had a significantly lower body weight at termination than those fed the control diet. Similar decrease in body weight has been observed previously in animals fed heme [26,35]. Even though we did not observe an increase in colonic lesions in animals fed hemin, the decreased body weight may indicate that there was a toxic effect of heme.

In conclusion, our results showed that dietary hemin decreased the number of colonic lesions in the A/J Min/+ mouse, an effect contrary to what was expected. However, the present study also revealed that dietary hemin had an opposite effect in the small intestine, where it appeared to stimulate tumor growth. Furthermore, our results showed that dietary nitrite had no effect in the colon of the A/J Min/+ mice, but a suppressive effect on CRC promotion in the small intestine.

## Supporting Information

S1 Data Number, load, and size of colonic and small intestinal lesions. (XLSX)

S1 Fig Colonic lesions of the A/J Min/+ mouse. Both A) flat ACF, and B) tumor, were observed at 10X magnification in an inverse light microscope.
(TIF)

S1 Table Proximate analysis of AIN-93M Control diet and standard maintenance diet RM1. (DOCX)
