# Supplementary figures and images for: Effects of Hemin and Nitrite on Intestinal Tumorigenesis in the A/J Min/+ Mouse Model
Source: PLoS One. 2015 Apr 2;10(4):e0122880. doi: 10.1371/journal.pone.0122880 (PMC4383626; doi:10.1371/journal.pone.0122880)

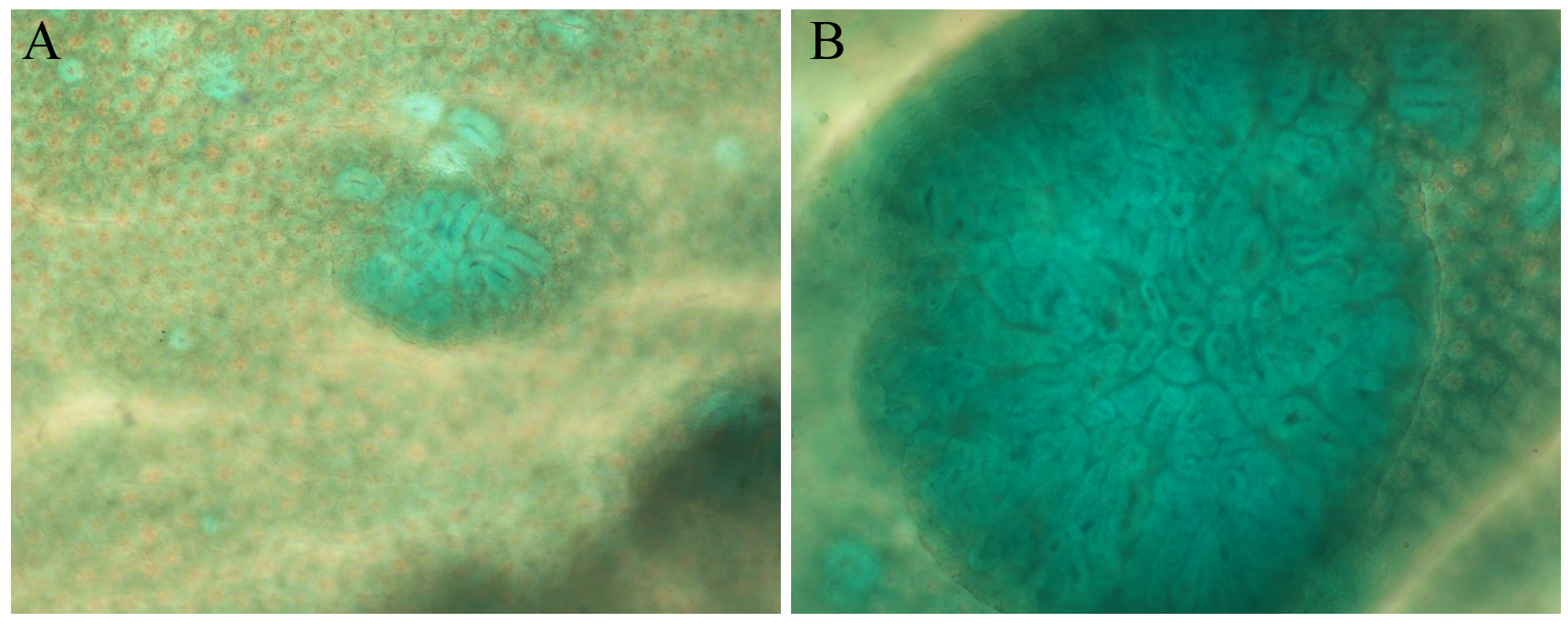

Supplement: S1 Fig — Both A) flat ACF, and B) tumor, were observed at 10X magnification in an inverse light microscope. (TIF) [file pone.0122880.s002.tif]
