# Supplementary material for: Effects of Hemin and Nitrite on Intestinal Tumorigenesis in the A/J Min/+ Mouse Model
Source: PLoS One. 2015 Apr 2;10(4):e0122880. doi: 10.1371/journal.pone.0122880 (PMC4383626; doi:10.1371/journal.pone.0122880)
Supplement: S1 Table — (DOCX) [file pone.0122880.s003.docx]

**S1 Table. Proximate analysis of AIN-93M Control diet and standard maintenance diet RM1.**

|  |  | AIN-93M | RM1 |
| --- | --- | --- | --- |
| Moisture | % | 10.00 | 10.00 |
| Crude Fat | % | 4.10 | 2.71 |
| Crude Protein | % | 11.60 | 14.38 |
| Crude Fiber | % | 3.31 | 4.65 |
| Ash* | % | 2.55 | 6.00 |
| Nitrogen Free Extract (NFE)^✝^ | % | 67.66 | 61.73 |

*Ash= total mineral or inorganic content; ^✝^NFE = all nutrients not included in previous categories, such as digestible carbohydrates, vitamins and other non-nitrogen soluble organic compounds [1].
Source: SDS special diet services, Witham, UK.

Reference

1. Olvera-Novoa MA, Martínez-Palacios CA, Real de León E (1994) Nutrition of fish and crustaceans a laboratory manual. Mexico City: Food and Agriculture Organization of the United Nations. Available: http://www.fao.org/docrep/field/003/AB479E/AB479E03.htm. Accessed 6 January 2015.
